# Super-resolved three-dimensional near-field mapping by defocused imaging and tracking of fluorescent emitters

**DOI:** 10.1515/nanoph-2022-0546

**Published:** 2022-10-24

**Authors:** Taehwang Son, Gwiyeong Moon, Changhun Lee, Peng Xi, Donghyun Kim

**Affiliations:** School of Electrical and Electronic Engineering, Yonsei University, 50 Yonsei-ro, Seodaemun-gu, Seoul, 03722, Korea; Department of Biomedical Engineering, College of Future Technology, Peking University, Beijing, 100871, China

**Keywords:** defocused imaging, emitter, near-field, tracking

## Abstract

Near-field optics is essential in many nanotechnology applications, such as implementing sensitive biosensing and imaging systems with extreme precision. Understanding optical near-fields at the nanoscale has so attracted the considerable research interest, which use a variety of analytical approaches, most notably near-field scanning microscopy. Here, we show defocused point localization mapped accumulation (DePLOMA), which can overcome many weaknesses of conventional analytical methods. DePLOMA is based on imaging fluorescence emitters at an out-of-focal plane. The acquisition, collection, and accumulation of the position and fluorescence intensity of emitters moving above nanostructures can generate three-dimensional near-field maps of light distribution. The idea enables super-resolution liquid-phase measurements, as demonstrated by reconstruction of near-field created by nanoslits with a resolution determined by emitter size. We employed fluorescent emitters with a radius of 50 and 100 nm for confirmation. The axial resolution was found to be enhanced by more than 6 times above that of diffraction-limited confocal laser scanning microscopy when DePLOMA was used.

## 1 Introduction

Near-field optics investigates excitation, management, and control of light on the wavelength scale [1]. Near-field distribution of light fields can be tailored in various approaches using, for example, plasmonic nanostructures [2–8], wavefront shaping [9, 10], and metamaterials [11, 12]. Tailoring near-fields has led to a wide range of applications that include bio-chemical sensing [13–19], optical tweezer [20–22], lithography [23–25], nonlinear optics [26, 27], data storage [28, 29], and subwavelength imaging [30–32].

In conjunction with the measurement of optical near-fields, localization microscopy has recently drawn significant attention. Localization microscopy was initially used for tracking positions of single fluorophores with nanometer-scale resolution [33] and applied further to produce super-resolution images by exploiting photochemical properties of dyes while sacrificing acquisition time [34, 35]. The concept has been extended to mapping surface electric fields [36–39] and also to attaining two-dimensional optical field distribution in the lateral plane without extra scanning procedure [40]. The technique has been implemented for the measurement of two-dimensional lateral fields induced by plasmonic nanogap [41], nanoslits [42] and silicon nanocavity [43]. One-dimensional axial optical field was also measured by holographic detection of metal particle scattering [44] and surface-enhanced Raman scattering from a plasmonic ruler [45].

Despite growing interests in near-field engineering, super-resolved acquisition of the nearfield distribution has remained relatively limited. Super-resolved surface mapping of near-fields is often performed with probe-based techniques such as near-field scanning microscopy (NSOM) [46]. Not only is it difficult for commercial NSOM to obtain near-fields in the aqueous environment [47, 48], the resolution of NSOM is also restricted by the probe aperture which NSOM uses for mechanical scanning. The aperture size is typically on the order of ∼100 nm and may incur sample damage due to the sample-to-aperture contact [49]. In the case of apertureless NSOM, illumination may not be modified so that applications are usually restricted to spectroscopy and fluorescence imaging [49]. Beside NSOM, optical field measurement has also been conducted by the far-field optics: for example, confocal laser scanning microscopy (CLSM) measures light intensity by a point detector through a pinhole while scanning a specimen [50]. On the other hand, 3D serial-sectioning microscopy similar to CLSM was performed using image reconstruction of the diffraction pattern based on wave optics model, although this may not be applied to the acquisition of non-radiative fields due to the inherent far-field nature [51].

In this paper, we explore a drastically different approach to address super-resolved surface mapping of three-dimensional near-field distribution by the parallel acquisition of fluorescent light fields. Here, we use near-fields in line with the terminology in NSOM as fields in the region from a nanostructure surface axially extended by a few microns [52]. Assume a fluorescent nanoparticle as an isotropic emitter: if one is located to be out of focus, ring patterns appear in the image plane due to diffraction [53]. The radius of the outermost ring is linearly related to the axial distance between observation and focal plane [54]. Based on this relationship, imaging and/or tracking of single fluorescence beads [55] or quantum dots [56] can be performed with nanometer precision. Out-of-focus diffraction rings of fluorescence were obtained for analysis with super-localized precision in 4D data, consisting of fluorescence intensity and 3D position.

The defocused ring-based tandem acquisition of fluorescence, which we call defocused point localization mapped accumulation (DePLOMA), is an approach similar in part to PAINT (Point Accumulation for Imaging in Nanoscale Topography) in the way that data acquisition is performed in the lateral plane using emitters on move [39]. The concept of DePLOMA has similarities with simultaneous localization and mapping (SLAM) in robotics and autonomous vehicles, where robotic or mobile agents acquire or update unknown environmental parameters while keeping track of the agents [57]. DePLOMA can perform acquisition of optical field distribution using conventional fluorescence microscopy without additional optical set-up or electronic devices. It may also be extended to the measurement of near-fields with multiple wavelengths. The spatial resolution of DePLOMA in the three-dimensional acquisition space is governed mainly by the emitter size. If we consider using emitters ranging 100 nm or smaller in size and noting that conventional microscopy suffers from poor resolution especially along the axial direction, it is obvious that the improvement can be drastic for axial resolution, while for the lateral plane it is comparable to other super-resolution techniques**.**

## 2 Methods and materials

### 2.1 Experimental confirmation of DePLOMA

**Figure 1:**
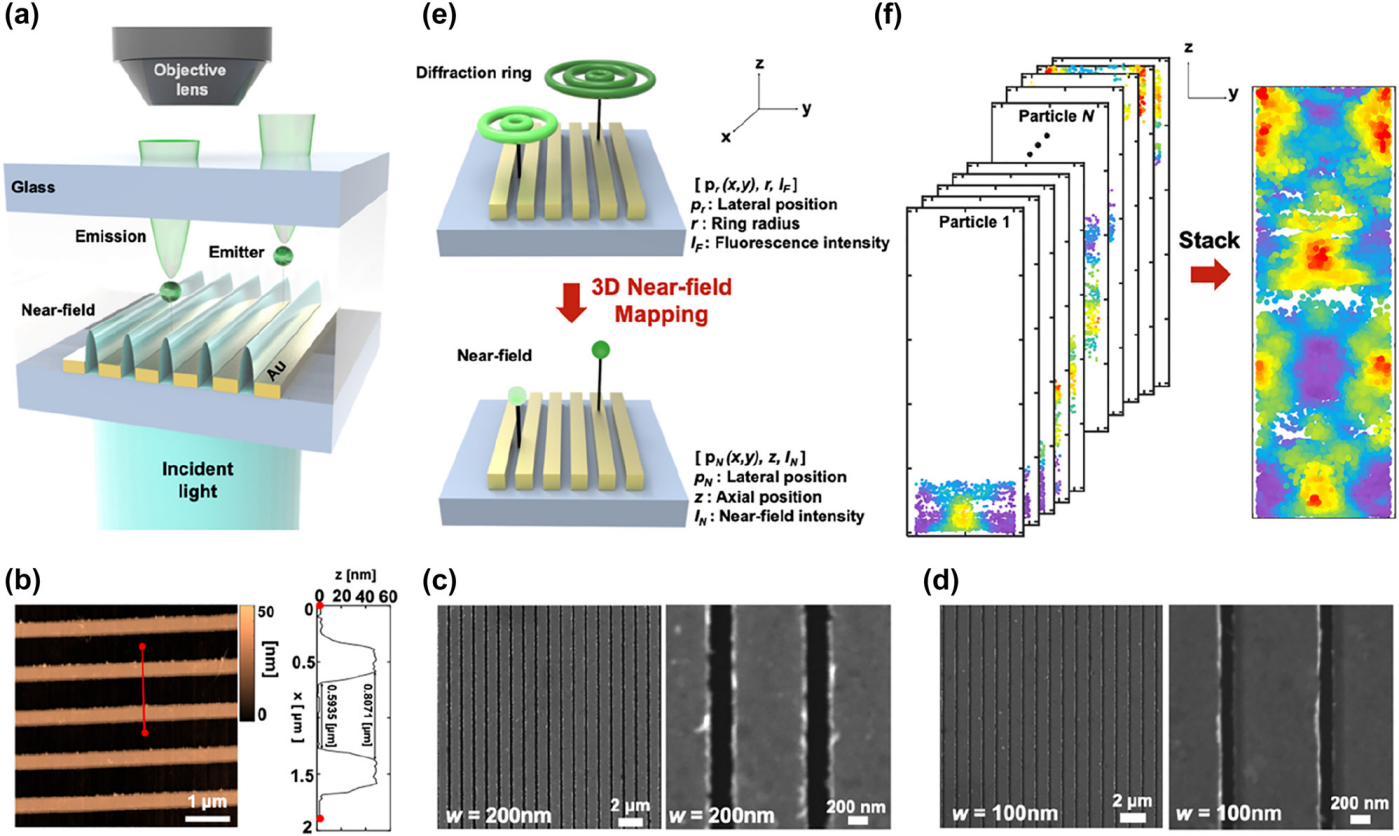
Conceptual schematics of DePLOMA. (a) Linearly polarized 488-nm plane wave light source is illuminated onto nanoslits. Sampling fluorescent beads create defocused ring patterns that are captured by the objective lens. Gold nanoslits located on the opposite side of illumination, which is sandwiched against another no. 1 glass coverslip, produce localized near-fields to be measured. The solution containing fluorescent emitters is used as an imaging medium. (b) AFM image and height profile of 700-nm width nanoslits (c) SEM image of 200-nm width nanoslits d SEM image of 100-nm width nanoslits (e) Near-field mapping from point accumulation derived from the diffraction ring patterns of fluorescent emitters, i.e., measured lateral position *p*_*r*_(*x*, *y*), ring radius *r*, and fluorescent intensity *I*_*F*_ are used to extract lateral position *p*_*n*_(*x*, *y*), axial position *z*, and near-field intensity *I*_*N*_ of fluorescent emitters. (f) Final near-field distribution was formed by stacking measurements from each fluorescent emitter.

The conceptual schematic of DePLOMA that we tested in this work is illustrated in Figure 1a. The nanoslit sample is placed on top of a glass coverslip with a droplet of solution containing fluorescent emitters as an adhesion layer. Without any mechanical or optical scanning, images of multiple randomly drifting or diffusing fluorescent objects were acquired simultaneously and translated into a map of optical field distribution in tandem. An axial map of optical field distribution can thus be created by accumulating diffraction ring images of fluorescent molecules in Brownian motion and drift. Three nanoslits with different widths (*w* = 700 nm, 200 nm, 100 nm) were employed for near field mapping. AFM image and height profile of 700-nm width nanoslits are presented in Figure 1b, and SEM images of nanoslits of 200-nm and 100-nm width are presented in Figure 1c and d, respectively.

### 2.2 Optical set-up

A commercial inverted microscope (IX-73, Olympus, Japan) was used for acquiring raw fluorescence images. The excitation light beam was manipulated to enter from the opposite side of an objective lens. A microscope motor stage and a nanostage (M-687, P-545, Physik Instrument, Germany) installed on the microscope body were used for coarse and fine position control. 488-nm laser (Coherent OBIS LX 50 mW) was expanded by a beam expander (GBE10-A, Thorlabs, USA) and aligned to the optical axis to be normally incident onto a nanoslit sample. An 60× (Olympus, UPlanFL N, NA 0.9) or 100× objective lens (Olympus, UApoN oil immersion TIRF lens, NA 1.49) was employed to place the focal plane 170–180 μm away from the objective lens. The focal plane produced by the objective lens was placed at the nanoslit surface by adjusting the correction collar at the objective lens. The images of fluorescent emitters in drift or Brownian motion were captured at 10 frames/second using an electron multiplying charge-coupled device (EM-CCD) (iXon Ultra 897, Andor), which is installed with a 2X magnification changer to reduce the size of a single pixel at an image plane. The schematic diagram for the optical setup is provided in Figure S1 in the Supporting Information.

### 2.3 Preparation of fluorescence emitter solution

We have used fluorescent beads as emitters. Fluorescent beads (FluoSpheres™ carboxylate-modified microsphere, yellow-green fluorescent (505/515), 2% solids, Thermofisher Scientific, USA, *f* = 100 and 200 nm) were diluted to 1:10^4^–1:10^5^ with DI water. To enhance signal-to-noise ratio (SNR) of fluorescence signals and to suppress Brownian motion, the solution was mixed with glycerol at a volume ratio of 1:4–6. As a result, the emitter concentration was controlled to be ∼1000 beads/mm^2^. The fluorescence intensity variation among emitters was tested using more than 100 emitter particles. The standard deviation and coefficient of variance in the measured intensity was obtained as 92.5 and 0.096 (Figure S2 in the Supporting Information).

### 2.4 Numerical simulation

DePLOMA was evaluated to capture optical near-fields produced by nanoslits. FDTD method was used to calculate the field distribution of nanoslits. Three different nanoslits were modeled with period (Λ), width (*w*), and height (*h*) with each parameter set to be (i) Λ = 1 μm, *w* = 700 nm, *h* = 50 nm, (ii) Λ = 1 μm, *w* = 200 nm, *h* = 50 nm, and (iii) Λ = 1 μm, *w* = 100 nm, *h* = 50 nm to form on chromium/gold films and BK7 glass substrate. Periodic and perfectly matched layer boundary condition were imposed along *x* and *z* direction, respectively. 488-nm linearly polarized plane-wave light source was assumed to be normally incident. Refractive indices of glass, chromium, and gold were taken as 1.5222, 1.056 + 1.0851*i*, and 2.3203 + 4.0253*i* [58].

### 2.5 Fabrication of nanoslits

For evaluation of DePLOMA, near-fields were produced by nanoslits formed in a 1D grating pattern. The nanoslits were fabricated by first cleaning a glass coverslip with acetone, isopropyl alcohol (IPA), and DI water. After spin-coating positive (AR-N 7520.073, Allresist, Strausberg, Germany) or negative resist (AR-N 7520.18, Allresist, Strausberg, Germany) at 4000 rpm, nanoslit patterns were defined by electron beam lithography. Finally, lift-off process was performed by depositing 50-nm thick gold film with a 2-nm chromium adhesion layer and removing resist. This creates gold nanoslit patterns of 50-nm height with a period Λ = 1 μm and various slit widths (*w* = 100, 200, and 700 nm).

### 2.6 Image processing

Image proceeding for DePLOMA was performed by custom-built MATLAB codes. Each Gaussian image of a fluorescent emitter was cropped from raw data. The least square fitting method was applied to estimate the coefficients of Gaussian ring patterns using a MATLAB function *lsqcurvefit.* Fluorescence intensity of an emitter was defined as the mean of the highest 100 pixel values in the cropped image.

## 3 Results and discussion

### 3.1 Axial calibration based on out-of-focus ring patterns

From the diffraction ring patterns of fluorescent emitters, 4-D information consisting of 3-D position and fluorescence intensity were acquired. The formation of defocused ring patterns of an isotropic emitter with respect to the axial distance from the focal plane is illustrated in Figure 1e. A diffraction ring can be produced above and below the focal plane due to diffraction of an imaging objective lens [55]. For convenience, the observation range below the focal plane, which is the closer to the objective lens, was selected for image acquisition. Figure 1f presents the schematics of measured data with a number of fluorescent emitters stacked together to form the final near-field distribution.

**Figure 2:**
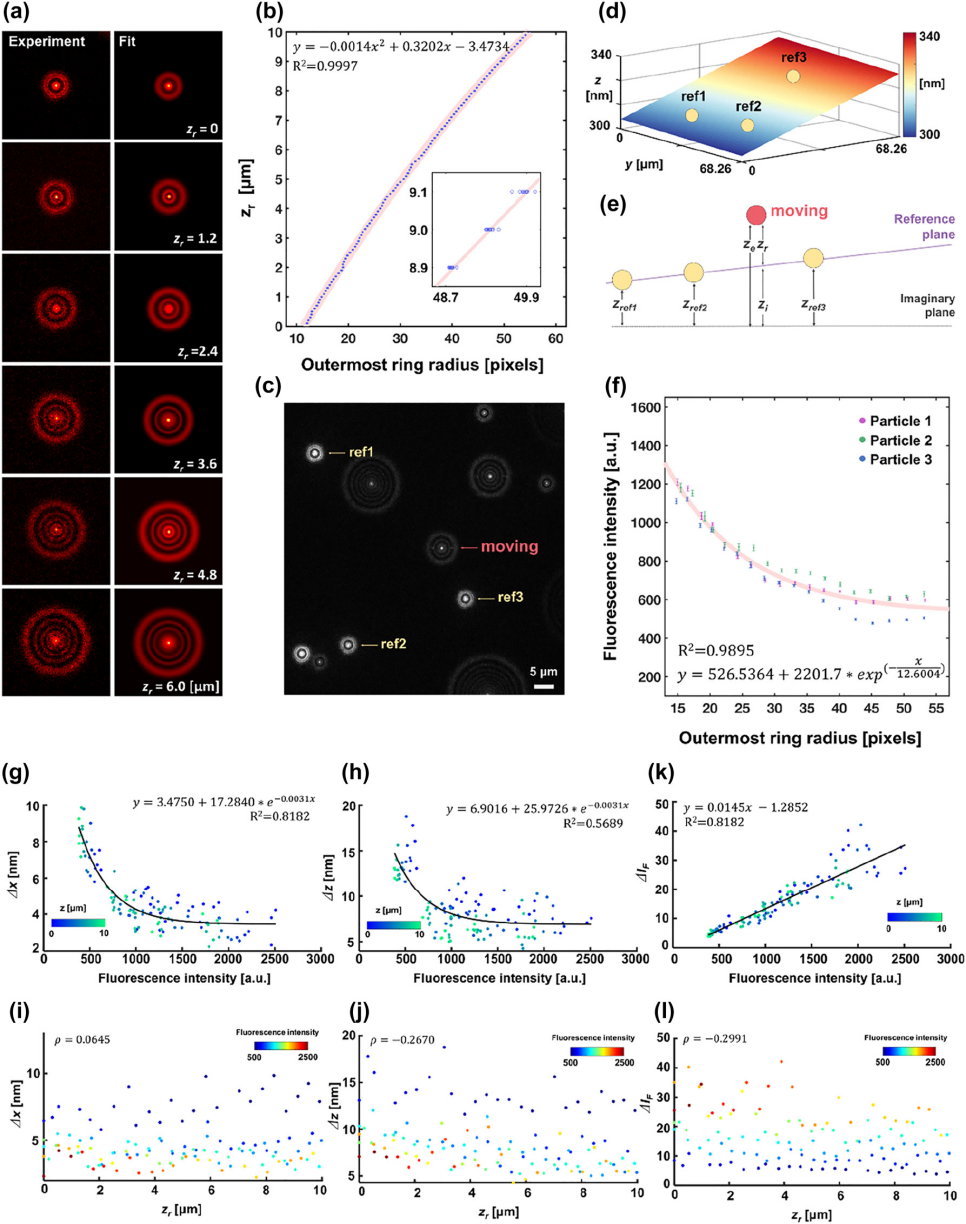
Calibration process of DePLOMA. (a) The diffracted images of a fluorescent bead (*ϕ* = 200 nm) at different axial positions *z*_*r*_ = 0–6 μm. (b) The relationship between the ORR and the *z*_*r*_. The second-order polynomial regression relation is also shown (*R* ^2^ = 0.9997). (c) A raw fluorescence image for DePLOMA where diffraction ring patterns from three static fixed reference fluorescent emitters (marked with yellow arrow) and one on the move (red arrow). (d) A plot of a plane formed with three fixed points defined by the positions of static fluorescent emitters (yellow circle). (e) Schematic diagram that presents the definition of various axial parameters: *z*_ref1_, *z*_ref2_, and *z*_ref3_ define a reference plane. *z*_*e*_ represents the axial distance of an emitter with *z*_*e*_ = *z*_*r*_ + *z*_*i*_. *z*_*i*_ is the distance from the imaginary plane to the reference plane and *z*_*r*_ from the plane to the emitter. (f) The relationship between *I*_*F*_ and the outermost radius (ORR) calculated with three fluorescent emitters. A curve fitted for calibration (*I*_calibration_) is also shown. (g, h) The relationship between fluorescence intensity (*I*_*F*_) and standard deviation of the measured emitter location along *x* and *z* axis (Δ*x* and Δ*z*). The solid line describes the fitted calibration function with a correlation coefficient *R* ^2^. (i, j) Relationship between relative axial position (*z*_*r*_) and standard deviation of the measured emitter location along *x* and *z* axes (Δ*x* and Δ*z*). (k) Linear relation is shown to be clear between average fluorescence intensity ( $\bar{I_{F}}$ ) and the precision of the measured fluorescence intensity (Δ*I*_*F*_). (l) The relationship between *z*_*r*_ and Δ*I*_*F*_ reveals that Δ*I*_*F*_ is independent of or very weakly correlated to the *z*_*r*_.

Defocused images for calibration were obtained by axially scanning the nanostage across a distance of 10 μm with a step of 100 nm, as presented in Figure 2a. Fluorescent emitters near nanoslits showed smaller ring patterns than those further away. Focal plane was adjusted so that the first ring should appear in defocused images of fluorescent emitter beads fixed on the nanoslits surface. The degree of focal plane adjustment was 5 and 2 μm away from the nanoslit, respectively, for the case using 60× (0.9 NA) and 100× (1.49 NA) objective lens. Use of a low NA objective lens improves stronger fluorescence intensity over a larger field-of-view. A high NA objective lens performs with enhanced lateral precision in the near-field mapping. For association of the acquired ring patterns with an axial position *z*_*e*_, they were fitted to a function *F* using Eq. (1), which consists of a Gaussian function with center (*x*_0_, *y*_0_) and width *w*_0_.

$$\begin{array}{ll} F\left(z\right) & =C_{0}+A_{0}\exp\left\{-B_{0}\left[{\left(x-x_{0}\right)}^{2}+{\left(y-y_{0}\right)}^{2}\right]/w_{0}{\left(z\right)}^{2}\right\} \end{array}$$

(1) $$\begin{array}{ll} & +\Sigma_{k=1}^{n}A_{k}\exp\left\{-B_{k}\left[{\left(x-x_{0}\right)}^{2}+{\left(y-y_{0}\right)}^{2}\right.\right.\\ & \;\left.\left.-R_{k}{\left(z\right)}^{2}\right]/w_{k}{\left(z\right)}^{2}\right\}, \end{array}$$

Concentric Gaussian rings share an identical center with different radii *R*_*k*_ and widths *w*_*k*_ (*n*: number of rings) where the outermost ring radius (ORR) is denoted as Rn. Figure 2b presents the calibration curve with an objective lens (NA = 0.9), in which the relationship between the ORR and the axial position (*z*_*e*_) is fitted to a second-order polynomial function. The ORR was expressed in image pixels and each pixel was 130 nm in size. Detailed procedures to obtain the axial position *z*_*e*_ from the ORR are presented in Supplementary Text in the Supporting Information. Overlapped two diffraction rings were distinguished and analyzed by considering the sum of two functions F1 + F2 [59] (Figure S3 in the Supporting Information).

For assessment of axial positions of fluorescent emitters in reference to the substrate surface, three-dimensional position of reference emitters was acquired to build a reference plane, as shown in Figure 2c. The diffraction ring patterns of three beads bound (ref1, ref2, ref3) to the surface were analyzed as reference positions. The reference planar surface formed by three points was obtained as a planar equation *f*(*x*, *y*) and the surface was represented in Figure 2d. The relative axial position (*z*_*r*_) of moving emitters with respect to the nanoslit surface was then obtained by subtracting the position of reference surface from that of fluorescent emitters, i.e., *z*_*r*_ = *z*_*e*_ − *z*_*i*_, where *z*_*i*_ is the value of *f*(*x*, *y*) at position of a moving emitter as represented in Figure 2e (Figure S4 in the Supporting Information) [60].

### 3.2 Fluorescence intensity correction

Fluorescence intensity *I*_*F*_ was defined as an average of the brightest 100 pixels in a diffraction image and then corrected by following an inverse square relationship between light intensity and ring radius. In order to minimize photobleaching that may affect the calibration between the ORR and fluorescent intensity, *z* position was axially scanned in a step larger than in Section 3.1 at 500 nm. For each axial position, 10 images were captured. The relation between fluorescence intensity and the ORR (*R*_*n*_) was calculated using three fluorescent beads as presented in Figure 2f, in which calibration was performed by fitting the curve with an exponential equation, *I*_calibration_ = *a* + b*exp(−*R*_*n*_/*c*). Calibrated fluorescence intensity is defined as *I*_*F,*cal_(*R*_*n*_) = *I*_*F*_(*R*_*n*_)/*I*_calibration_(*R*_*n*_).

### 3.3 Precision of fluorescence bead localization and intensity measurement

We estimated the localization precision from standard deviations of a fixed nanoparticle, for which fluorescence intensity *I*_*F*_ as well as the relative axial position *z*_*r*_ of a fluorescent emitter was varied by adjusting input laser power in the range of 0.533–7.840 W/cm^2^ and moving a sample stage axially. The relationship between *I*_*F*_ and standard deviation of the measured emitter location along *x* and *z* direction (Δ*x* and Δ*z*) is addressed in Figure 2g and h. Here, Δ*x* and Δ*z* are defined as the position of an emitter in each image frame relative to an average over the whole frames, i.e., Δ*x* = $x-\bar{x}$ and Δ*z* = $z_{r}-\bar{z_{r}}$ , and fitted into the calibration function. It is found that Δ*x* and Δ*z* decrease exponentially with higher fluorescence intensity of emitters due to improved signal-to-noise ratio (SNR). Also, Δ*x* is smaller than Δ*z* at a given *z*_*r*_, which implies that lateral localization precision is better than axial localization precision.

Figure 2i and j shows that the localization precision, or position error in the *x* and *z* direction, is little affected by the axial position of an emitter in the range of 0–10 μm as the Pearson’s correlation coefficient (*ρ*) of *x* and *z* is calculated to be 0.0645 and −0.2670, respectively. Average values of Δ*x* and Δ*z* are determined as *σ*_*x*_ = 3.95 nm and *σ*_*z*_ = 7.74 nm, suggesting sub-10 nm localization precision of the DePLOMA system. The localization precision may be affected by the process of fitting the diffraction pattern with the Gaussian ring function and can decline as a result of digitization error in the image acquisition, sample drift or vibration in the experimental set-up. The localization precision may also be influenced by the way that an emitter moves. Use of glycerol mixture as medium tends to suppress Brownian motion without aggravating the precision. On the other hand, the movement of emitters may produce defocused image blur and deteriorate localization error. The effect appears to be stronger along the axial direction than in the lateral plane and may contribute an additional 3 nm in the localization error.

The precision of fluorescence intensity (Δ*I*_*F*_) was obtained from the standard deviation of measured fluorescence intensity *I*_*F*_(*z*) of a fluorescent emitter while adjusting laser power along the *z* direction. The relationship between average fluorescence intensity ( $\bar{I_{F}}$ ) and Δ*I*_*F*_ is shown in Figure 2k. Δ*I*_*F*_ was found to be linearly proportional to $\bar{I_{F}}$ , because of the poor stability and intensity noise at high laser power [61]. On the other hand, the relationship between *z*_*r*_ and Δ*I*_*F*_ presented in Figure 2l confirms that Δ*I*_*F*_ is independent of or very weakly correlated to *z*_*r*_.

### 3.4 Tracking fluorescent emitters over nanoslits

**Figure 3:**
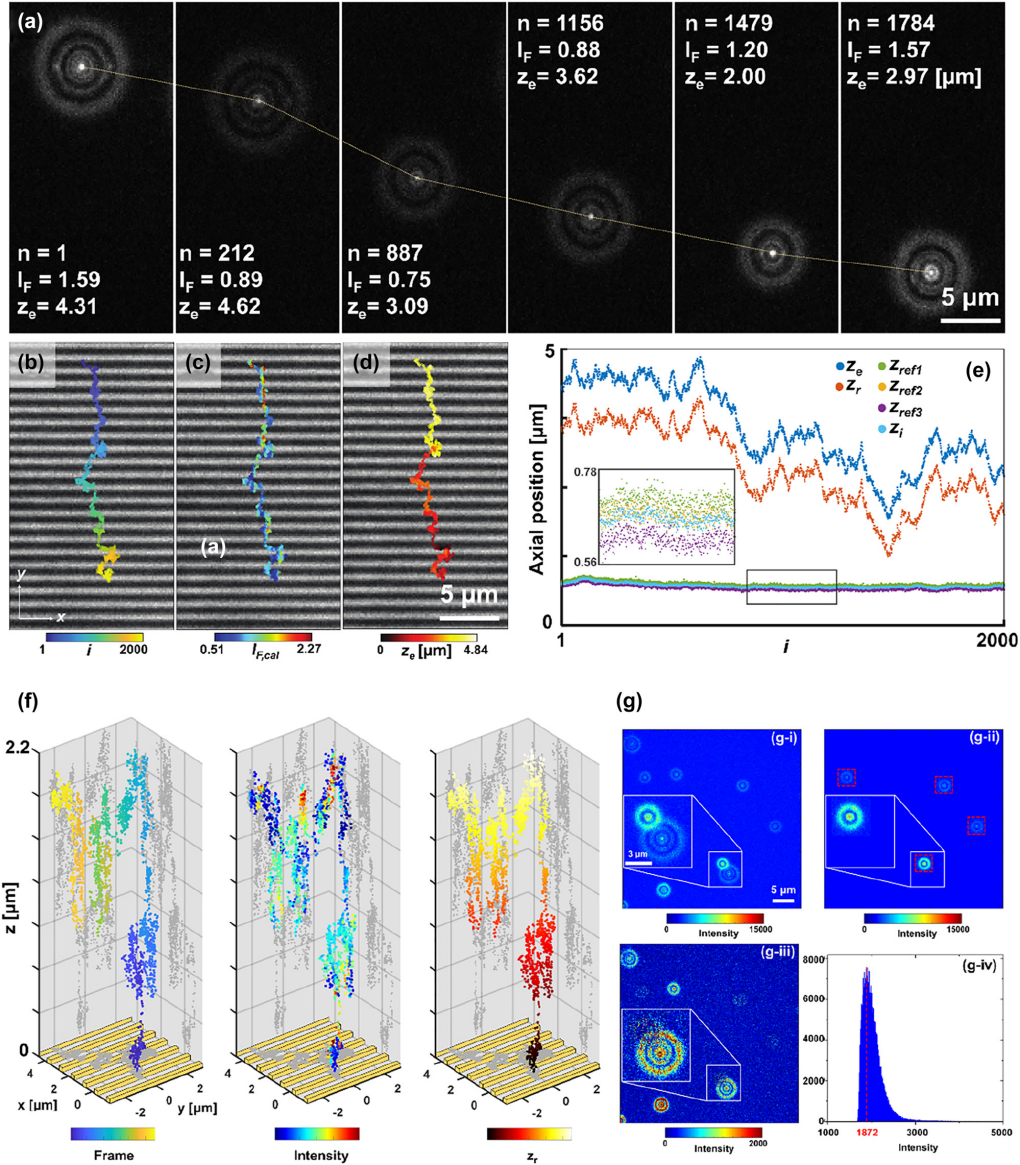
Three-dimensional tracking of fluorescent emitters. (a) Diffraction ring patterns of a fluorescent emitter above nanoslit arrays (frame index *i* = 1, 212, 887, 1156, 1479, and 1784). (b–d) Trajectories of fluorescent emitters near nanoslits for 2000 frames overlaid with a bright-field image of nanoslits. Color bars: frame number, fluorescence intensity and axial position *z*_*e*_. (e) Plot of *z*_*e*_, *z*_*r*_, and *z*_*p*_ as well as axial position of three reference emitters (*z*_ref1_, *z*_ref2_, *z*_ref3_) with respect to the frame number. (f) 3D trajectories of a fluorescent emitter. Three different color maps represent the frame number (left), fluorescence intensity (middle), and relative axial position (right). (g) Representative overlapped two ring image induced by adjacent two emitters. (i) a raw image, (ii) a reference image, and (iii) a difference image. (iv) Intensity histogram of (g–i).

Based on the schematic illustrated in Figure 1, fluorescent emitters excited by the near-field of nanoslits were tracked in DePLOMA using the optical set-up presented in Figure 1a. Figure 3a shows the images of diffraction ring patterns produced by fluorescent emitters in glycerol solution. The Brownian motion of fluorescence emitters was suppressed in the glycerol mixture to improve the SNR of fluorescence signals. Analysis of correlation between electrical field gradient and bead movement suggested the effect of optical trapping force on the movement of a fluorescent emitter near the plasmonic nanoslits to be negligible. The intensity of the ring patterns, although depending partly on the incident light power fluctuation, is mainly determined by the optical near-fields produced by nanoslits. These ring images can be converted to the trajectory of a fluorescent emitter moving over nanoslits for each frame, as shown in Figure 3b, which is overlaid with a bright-field image at the same position (Figure S5 in the Supporting Information). Color bar presents frame number, fluorescence intensity, and axial position *z*_*e*_, respectively. In particular, Figure 3c presents the fluorescence intensity of an emitter *I*_*F,*cal_(***r***, *t*), which is corrected by fluorescence intensity calibration described in *Fluorescence intensity correction*. The axial position of an emitter was obtained from the calibration curve by fitting each image frame to the function *F* in Eq. (1) and thereby determining the ORR (*R*_*n*_), as shown in Figure 3d. The axial movement of this emitter is described in Figure 3e, in which the axial variation of *z*_*i*_, *z*_*e,*_ and *z*_*r,*_ as well as three reference emitters (z_ref1_, z_ef2_, and *z*_ref3_) are presented. The inset of Figure 3e shows magnified axial position of the three reference emitters as well as *z*_*i*_. The results show clearly that the moving emitter under observation tends to travel over a fairly long axial distance in a direction, in general, toward the surface with an average speed *v*_avg,*z*_ = 516 nm/s measured in 200 s (Figure S6 in the Supporting Information). Figure 3f shows reconstructed trajectories of a fluorescent emitter, which dynamically moved around the nanoslit in the 3D space. The results suggest dramatical intensity variation according to the location. Note that the fluorescent emitters described in Figure 3a–d and Figure 3f are not identical. Overall procedure of near-field mapping is described in Supplementary Movie 1. We developed an algorithm which fits two ring patterns at the same time by two Gaussian ring equations if multiple emitters in a frame were close enough so there is chance of overlapping. Especially, in order to avoid the overlap between moving fluorescence emitters and reference emitters, difference images (*I*_dif_, Figure 3g-iii) were obtained by subtracting a raw image (*I*_raw_, Figure 3g-i) from a reference image (*I*_ref_, Figure 3g-ii). While leaving the cropped area of reference emitters (red dashed squares in Figure 3g-ii), the intensity values of rest area were replaced by the most frequent value of raw image, which can be obtained from the intensity histogram (Figure 3g-iv). More details are provided in Figure S3 in the Supporting Information. Through this procedure, we can obtain the reference bead image from the overlapped image. Therefore, the difference extracts a diffraction image of moving fluorescence beads, which relieves possible fitting errors aroused from the crosstalk between reference beads and moving beads.

**Figure 4:**
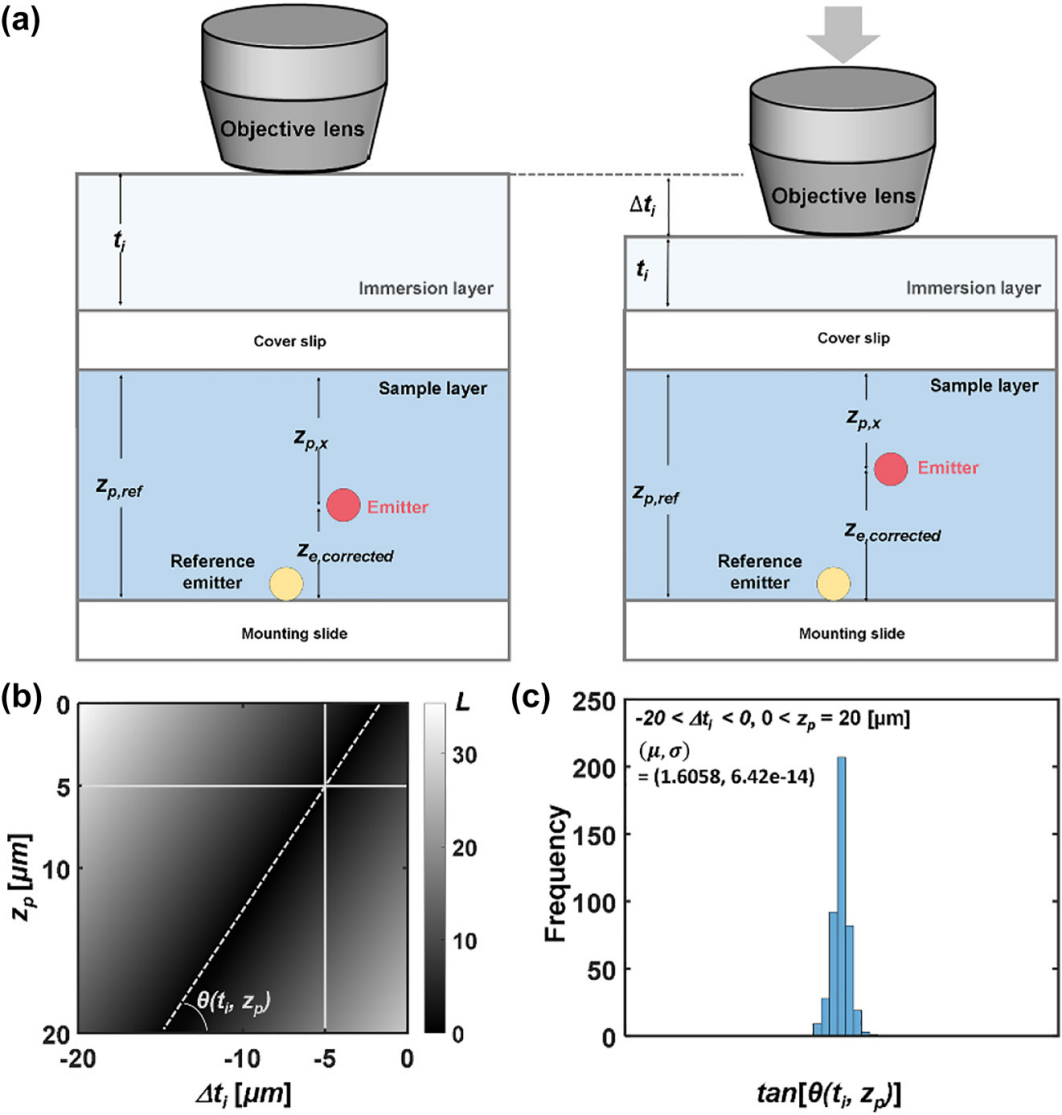
Compensation of refractive index mismatch between immersion layer and water/glycerol solution. (a) Schematic illustration of experimental conditions: a fluorescent emitter positioned in a sample layer in refractive index distribution. Downward movement of an objective lens reduces *t*_*i*_ (Δ*t*_*i*_ < 0). Refractive index of the immersion and the sample layer: *n*_*i*_ = 1.0 and *n*_*s*_ = 1.4471. (b) Variance measure *L* was obtained in the range of −20 μm < Δ*t*_*i*_ < 0 and 0 < *z*_*p*_ < 20 μm with respect to (Δ*t*_*i*_, *z*_*p*_) = (−5 μm, 5 μm). The angle formed by linear contour and horizontal axis is defined as *θ.* (c) The distribution of the angle *θ* in the range of −20 μm < Δ*t*_*i*_ < 0 and 0 < *z*_*p*_ < 20 μm.

Additional axial correction is necessary as described in Figure 4a to compensate for the refractive index mismatch between the immersion layer and water/glycerol solution, i.e., optical path lengths (OPLs) in fixed probe imaging and of the tracking probes in Brownian motion are disparate. The distance between the objective lens and the cover glass induces OPL change in the axial calibration process, while the movement of a probe leads to a change in the OPL during the tracking of probes in motion [62, 63]. We used the Gibson and Lanni model to calculate the axial correction factor [64, 65], where the point spread function (PSF) of a fluorescent emitter is determined by refractive indices of an immersion layer and a sample layer. The emission wavelength of fluorescent emitters was 515 nm with NA = 0.9. *n*_*s*_ and *n*_*i*_ are the refractive indices of water/glycerol solution and air, i.e., *n*_*s*_ = 1.4471 and *n*_*i*_ = 1 [66]. For comparison of OPLs before and after correction, a measure of variance *L* is introduced when Δ*t*_*i*_ and *z*_*p*_ are given. More details of the procedure leading to the definition of *L* are provided in Figure S7 in the Supporting Information.

Figure 4b shows *L* in the 2D plane along the Δ*t*_*i*_ and *z*_*p*_ axis with reference to (−5 μm, 5 μm). In this case, *L* = 0 if (Δ*t*_*i*_, *z*_*p*_) = (−5 μm, 5 μm). Because the contour of *L* is linear, *L* = 0 on a line that passes (−5 μm, 5 μm) with an identical ORR. If we define the angle that the linear contour makes with respect to the horizontal axis as *θ*, the distribution is plotted in Figure 4c. In the range of Δ*t*_*i*_ and *z*_*p*_ encompassing experimental conditions, tan *θ* is determined to be 1.606. The position of a moving emitter relative to the reference bead can thus be obtained by multiplying the axial path length difference with tan *θ*, and more details are provided in Figure S7 in the Supporting Information. In other words, the correct axial position (*z*_*e,*corrected_) can be obtained by simply multiplying tan *θ* as the correction factor *cf*, i.e., *z*_*e,*corrected_ = *cf* × *z*_*e*_. Tolerance analysis assuming deviation in the sample refractive index *n*_*s*_ suggests that the effect on the near-field mapping is rather limited, i.e., Δ*n*_*s*_ = 0.01 leads to a deviation of 0.82% in *cf* and *z*_*e,*corrected_.

### 3.5 Fluorescence intensity versus optical field

Since we have obtained 3D positions of fluorescent emitters, we now figure out the relation between measured fluorescence intensity and optical near-fields. In principle, excited fluorescence intensity, if unsaturated, is linearly proportional to the incident light power [67]. The linear relationship between fluorescence and optical near-field intensity has been applied to the confirmation of near-field enhancement from a gold nanoparticle due to plasmon-to-fluorescence energy transfer using fluorescent beads as an indicator [68, 69]. The linearity of the relationship was experimentally confirmed by measuring fluorescence intensity while varying input laser power. Figure S8 in the Supporting Information presents the fluorescence intensity of diffraction ring patterns at *z* ∼ 5 μm away from the surface. The results show that fluorescent intensity grows linearly as the input laser power increases from 0 up to 2.04 W/cm^2^ (*R* ^2^ = 0.99) and then starts to saturate. Therefore, fluorescence intensity *I*_*F,*cal_ was acquired in the linear region and simply converted to the optical field intensity in the unit of W/cm^2^. Potentially adverse fluorescence bleaching was also checked and found to be negligible under the experimental conditions (Figure S9 in the Supporting Information).

Fluorescence intensity is directly influenced by the axial and lateral movement of a fluorescent emitter in the optical near-field. Interestingly, fluorescence intensity trajectories presented in Figure 3c exhibit strip patterns and were found to be correlated with the background bright-field image of nanoslits. Horizontal bright lines along the *x* direction refer to the location of gold slits acquired in the back-reflected bright-field image. Also, note that movement orthogonal to nanoslits (*y* direction) incurs more significant changes in light intensity than the one along the parallel (*x* direction) or axial direction (*z* direction) due to steeper changes in the optical field distribution across nanoslits.

### 3.6 Near-field mapping

**Figure 5:**
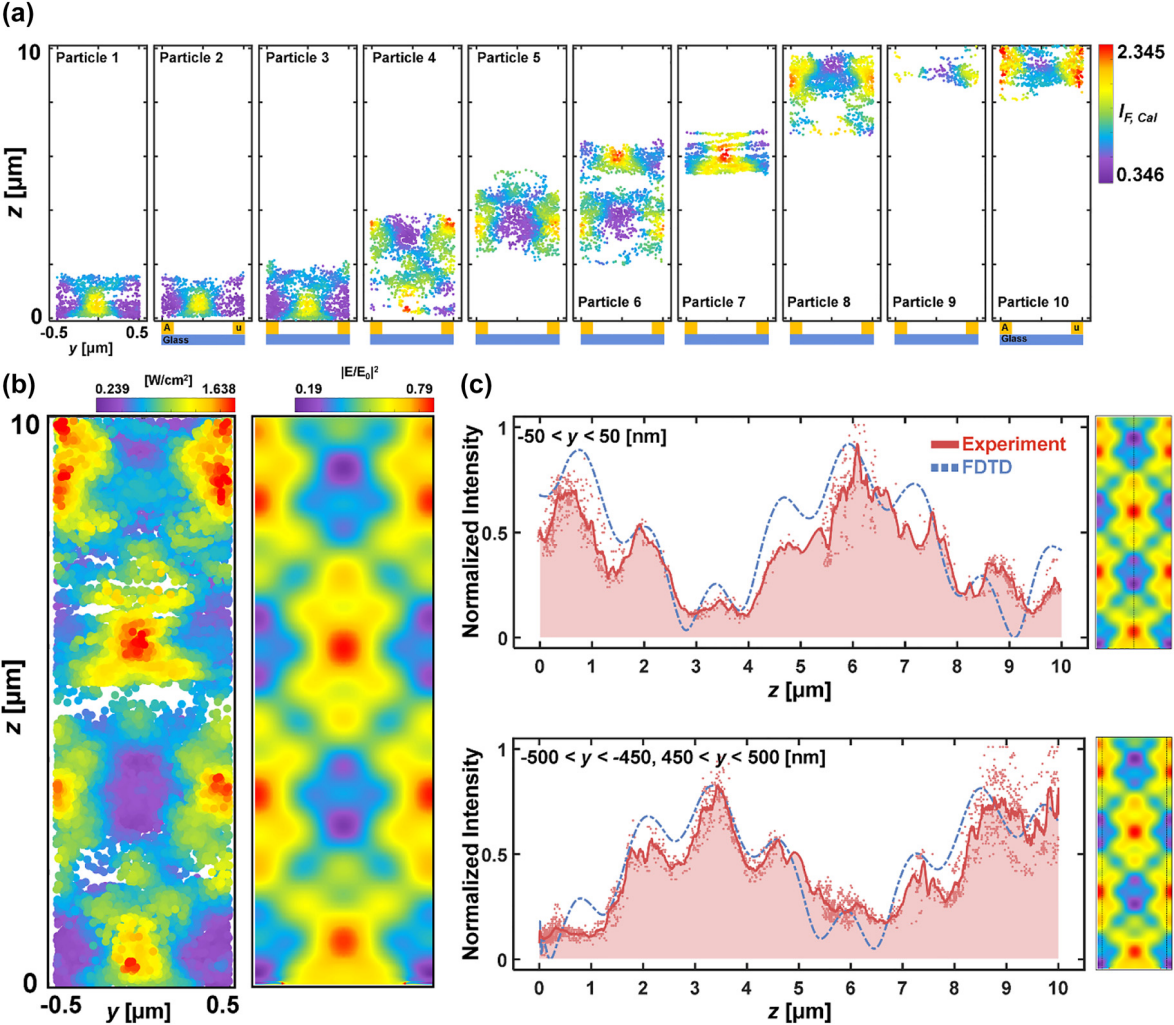
Near-field mapping over 700-nm width of nanoslits. (a) Individual near-field component maps in the *yz* plane obtained with 8 fluorescent nanoparticle emitters. (b) Left: merged optical near-field intensity map out of 14 fluorescent emitters. Right: theoretical near-field distribution induced by nanoslits. Both were evaluated at excitation wavelength (*λ* = 488 nm). (c) Normalized intensity profiles of *I*_*F,*cal_ along *y =* 0 (top) and *y =* 0.5 µm (bottom) presented in block-dotted lines. Points within a 50-nm distance were projected to form a line in the *z*-direction. The theoretical near-fields are given in blue solid lines for comparison.

For confirmation of DePLOMA, we used fluorescent emitters as probes to sample and measure localized near-fields that are produced by nanoslits. Optical near-fields obtained by 200-nm fluorescent nanoparticle emitters moving along the *y* direction are projected in the *yz* plane to build a near-field intensity map over a single nanoslit period. Near-field maps created by 8 independent fluorescent emitters are shown in Figure 5a. More near-field maps from fluorescent nanoparticles are presented in Figure S10 in the Supporting Information. Reduction of the speed of fluorescent nanoparticles due, for example, to the mixture of glycerol leads to improved SNR in the near-field mapping. Statistics for the velocity of fluorescent nanoparticles in the lateral plane and along the depth axis is presented in Figure S11 in the Supporting Information. Individual near-field component maps out of 14 fluorescent emitters were merged to produce an overall near-field distribution, which consists of 21,235 measurement points in total and agrees well with a theoretical near-field distribution as presented in Figure 5b. Near-field distribution was also calculated at emission wavelength (*λ*_em_ = 515 nm) and presented in Figure S12. The nanoslit position was registered by fitting bright-field images to sinusoidal equations (see Figure S5 in the Supporting Information). Figure 5c shows the fluorescence intensity *I*_*F,*cal_ profiles along *y* = 0 (top) and *y* = 0.5 µm (bottom). Note that the period of nanoslits is 1 µm. The scatter plots in black were generated by projecting points within a 50-nm distance in the *z*-direction. The plots represent raw data without any interpolation. Based on the results described in *Precision of fluorescence bead localization and intensity measurement*, the localization precision of the near-field distribution in Figure 5b was estimated to be 2–10 nm in the lateral plane and 5–20 nm axially (see Figure S13 for the data).

**Figure 6:**
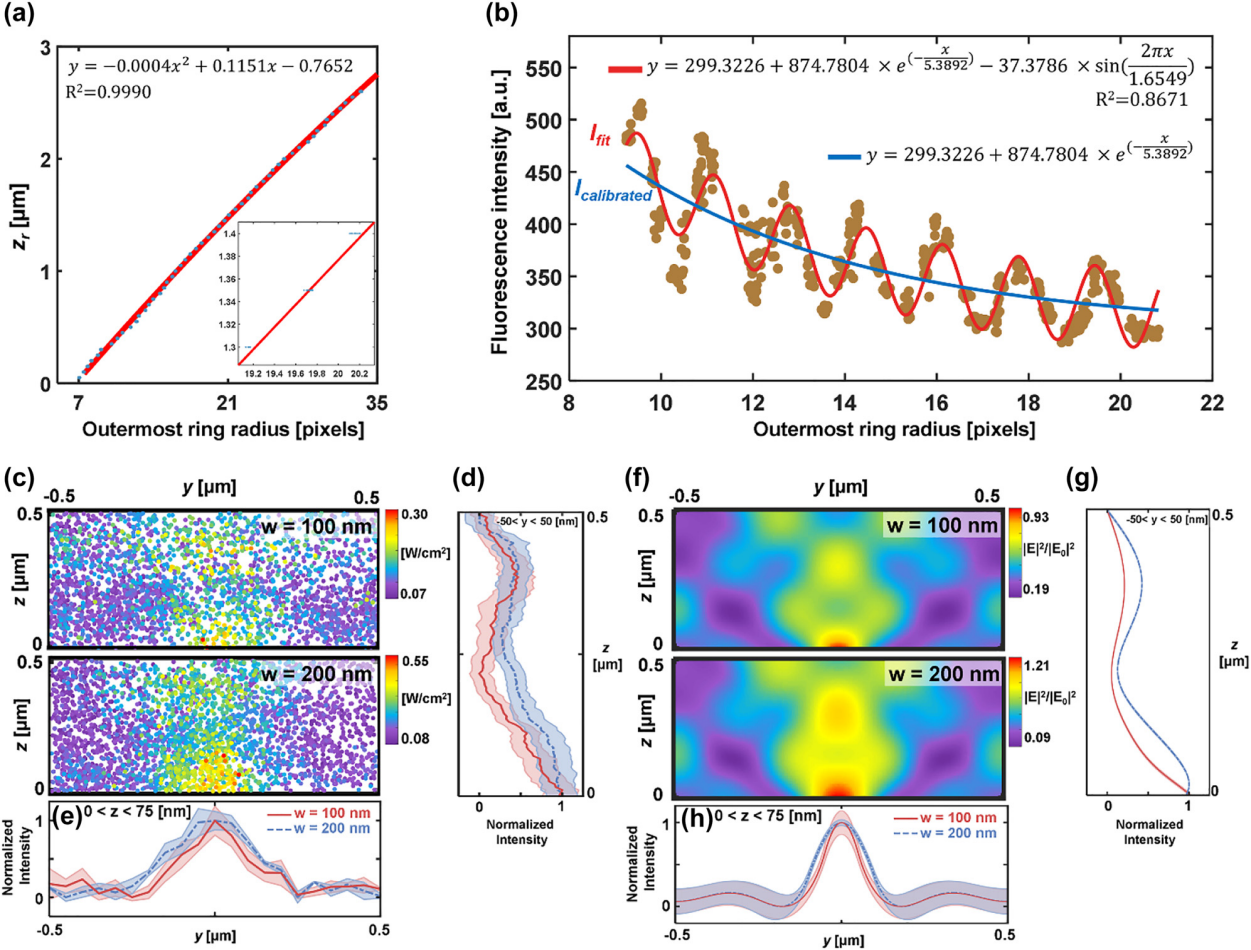
Near-field mapping over 100-nm and 200-nm width of nanoslits. The relationship of the ORR versus (a) relative axial position and (b) fluorescence intensity with a NA 1.49 objective lens. The dotted experimental values are fitted by the function shown in each figure. Inset: magnified section suggesting a linear fit. (c) DePLOMA of near-fields produced by nanoslits (*w* = 100 and 200 nm). Mapping was performed with fluorescent emitters of 50-nm radius. Near-field intensity map was constructed by merging data measured by 22 fluorescent emitters. (d) Normalized intensity profile of *I*_*F,*cal_ along *y*-axis for nanoslits with *w* = 100 nm (red) and 200 nm (blue). The points with *z* value from 0 to 75 nm were projected to the *y*-direction. The colored shade represents the standard deviation in the intensity along the *y* direction. Normalized intensity profiles of *I*_*F,*cal_ along *z*-axis for nanoslits with *w* = 100 nm (red) and 200 nm (blue and dashed). The points within a 50-nm distance were projected to the *z*-direction. Colored shade represents the standard deviation in the intensity along the axial direction. (f) Theoretical near-field distribution produced by nanoslits (*w* = 100 and 200 nm). (g) Normalized intensity profile of near-field distribution along *y*-axis for nanoslits with *w* = 100 nm (red) and 200 nm (dashed blue and dashed). The points with *z* value from 0 to 75 nm were projected to the *y*-direction. (h) Normalized intensity profiles of near-field distribution along *z*-axis for nanoslits with *w* = 100 nm (red) and 200 nm (dashed blue and dashed). The points within a 50-nm distance were projected to the *z*-direction.

Mapping was performed with 100-nm fluorescent emitters to measure near-fields produced by nanoslits of 100 and 200-nm width. The diffraction of fluorescence was imaged adjusting the focal plane of NA 1.49 objective lens. Sample medium was water/glycerol solution with the refractive index is 1.4555 [66]. The dynamic range as the maximal observable axial distance is approximately 8 μm (NA = 1.49), which may be affected by the lateral emitter position off the center of diffraction rings. Figure 6a shows the relation between relative axial position (*z*_*r*_) and ORR which is fitted to a second-order polynomial function. Note that the calibration curve of Figure 6a was obtained with an objective lens NA = 1.49 for a pixel size of 0.08 μm. Fluorescence intensity (*I*_*F*_) versus the ORR is also presented in Figure 6b. I_*F*_ overall decreases while varying periodically with a period of 1.655 pixels in the ORR. The period can be translated to 0.162 μm in *z*_*r*_, which corresponds to the variation of thickness of immersion layer caused by axial scanning. This periodic variation of *I*_*F*_ can also be explained by Fabry–Perot effect between bottom glass and objective lens: interference occurs between the two layers and the fluorescence intensity varies with respect to the thickness of immersion layer. The period then is given by *λ*/2*n*_*i*_ = 0.162 μm in perfect agreement with the experimental data. Actual intensity correction was performed with *I*_calibration_ (blue line in Figure 6b) excluding the sinusoidal term considering the fixed thickness of an immersion layer while tracking fluorescent emitters.

Optical near-fields obtained by 100-nm fluorescent emitters moving along the *y* direction are projected in the *yz* plane to build a near-field intensity map over a single nanoslit period. Near-field maps from each fluorescent emitter are presented in Figure S14 in the Supporting Information. Figure 6c shows the map of near-fields produced by 100 and 200 nm nanoslits which consists of 258 and 405 measurement points, respectively, which is in good agreement with theoretical results presented in Figure 6f. Figure 6d and e show the normalized intensity profiles of *I*_*F,*cal_ along *x* = 37.5 nm and *y* = 0 for nanoslits with *w* = 100 nm (red) and 200 nm (dashed blue). The line profiles were obtained by projecting measured points within a 37.5 nm distance in the *y*-direction (Figure 6d) and a 50-nm distance in the *z*-direction (Figure 6e), respectively. Note that Figure 6g and h were obtained in a similar manner as Figure 6d and e by averaging data within a 37.5 nm distance in the *y*-direction (Figure 6g) and a 50-nm distance in the *z*-direction (Figure 6h), respectively. DePLOMA produces low-pass filtered field distribution compared to the one calculated by FDTD simulation shown in Figure 6f–h, because the reconstruction by DePLOMA represents an average intensity over a volume defined by the finite size of an emitter. The emitter size also affects the resolution of DePLOMA, as discussed below. The increase in the width of localized field reconstructed by DePLOMA in comparison to theoretical results may be associated with imperfect nanoslit fabrication and polarization alignment errors.

Note that fluorescent emitters moving outside the proximity of metal structure, typically beyond 20 nm from the surface, were used as near-field probe to measure optical field intensity and to avoid distortion due to photon–plasmon energy transfer. The approach can be extended to other complex 3D structures such as periodic nanoholes, nanopillars, and nanodisks that produce different near-field intensity patterns. Current least-square-regression image analysis has a limitation on the number of emitters that can be simultaneously processed or overlapped in a field of view. Note also that application of DePLOMA may be extended to mapping near-fields produced by complex 3D nanostructures by deep learning-based diffraction ring pattern analysis due to its robustness in noise reduction and pattern recognition [70, 71]. Deep learning-based analysis can address limitations using massive training sets generated from both experimental and theoretical point spread function model. The number of emitters can be optimized, e.g., by the integration of microfluidic channels with an automated flow system to place bead emitters appropriately on the region of interest.

As a performance measure of DePLOMA, the effect of an emitter size is worth to discuss: the resolution is dependent directly on the size of fluorescent emitters that scan the fields under measurement, i.e., the size correlates with the width of the point spread function in optical microscopy. This is because any two targets displaced from each other by more than the emitter size can be resolved (Figure S15 in the Supporting Information). For this reason, the use of a smaller emitter should improve the resolution both in the lateral and the axial dimension at the expense of SNR. Because of isotropic emission in all directions, the effective resolution is also isotropic; therefore improvement in the axial resolution should be much more significant than in the lateral plane. For example, use of nanoparticle emitters of 100-nm radius provides moderate improvement over conventional wide-field microscopy in the lateral resolution since the diffraction-limited lateral resolution is 286 nm with NA = 0.9 and *λ*_em_ = 515 nm. However, improvement in the axial direction can be quite significant: if compared with wide-field axial diffraction limit of 1272 nm, axial resolution is improved by 6.4 times (=1272/200) [72]. With respect to CLSM, axial resolution is given by 1.4*n*_*s*_ *λ*/NA^2^ = 890 nm, i.e., the improvement of axial resolution by DePLOMA becomes 4.5 times with emitters of 100-nm radius.

The resolution of DePLOMA may be improved by introducing a smaller emitter such as a quantum dot at the expense of additional optical complexity and sophisticated analysis algorithm to overcome limited photon acquisition [53]. If emitters are too small, e.g., single molecule dyes, the number of acquired photons may be insufficient to produce ring patterns at video rate, thus implementation of acceptable temporal resolution and image acquisition with good SNR becomes difficult. In this sense, an optimum size of an emitter probe should exist, which we estimate between 10 and 100 nm at video rate.

## 4 Conclusions

In summary, we report DePLOMA based on point mapping and accumulation. Defocused images allow fluorescent emitters to be localized for acquisition and mapping of the near-field. Data accumulated leads to 3D image construction of the near-field distribution produced by nanostructures. Image resolution is directly connected with the size of an emitter. The concept was confirmed by measuring near-fields formed by nanoslits of 100 and 200-nm width. With nanoparticle emitters of 50-nm radius, significant enhancement of resolution was observed along the axial direction by more than 6 times while it was moderate in the lateral plane.

The main advantage of DePLOMA is the capability of extracting super-resolved information with the compatibility to an aqueous environment, because DePLOMA uses floating the fluorescence emitters in liquid medium. Also, DePLOMA measures both propagating and evanescent field while confocal laser scanning microscopy may only measure propagating fields. This way, DePLOMA can achieve improved lateral and axial resolution, as confirmed experimentally in this work. On the other hand, fluorescent beads move randomly over target nanostructure without external control, preventing efficient data acquisition, which combined with desired use of a single fluorescent bead increases the image acquisition time. In the future, control of fluorescent beads by optical trapping techniques is expected to allow more efficient data acquisition by reducing measurement time. Point spread function engineering can be adopted for reconstruction of field maps in the wide axial range Use of smaller fluorescent particles may enhance lateral and axial resolution of DePLOMA. Also, deep learning-based image processing can replace the least-square-regression analysis which has the limitation on the number of fluorescence emitters to process in a single image.

## Supplementary Material

Supplementary Material Details
