# Supplementary material for: Super-resolved three-dimensional near-field mapping by defocused imaging and tracking of fluorescent emitters
Source: Nanophotonics. 2022 Oct 24;11(21):4805–19. doi: 10.1515/nanoph-2022-0546 (PMC11501887; doi:10.1515/nanoph-2022-0546)
Supplement: Supplementary file 1 — Supplementary Material Details [file j_nanoph-2022-0546_suppl_001.docx]

**Super-resolved three-dimensional near-field mapping by defocused imaging and tracking of fluorescent emitters**

**Supplementary Information**

Taehwang Son^[[1]](#footnote-2)^^[[2]](#footnote-3)1^, Gwiyeong Moon^†1^, Changhun Lee^[[3]](#footnote-4)1^, Peng Xi^2^ & Donghyun Kim^1*^

^1^School of Electrical and Electronic Engineering, Yonsei University, 50 Yonsei-ro, Seodaemun-gu, Seoul, Korea, 03722

^2^Department of Biomedical Engineering, College of Future Technology, Peking University, Beijing, 100871, China
Corresponding author: kimd@yonsei.ac.kr.

| **Figure S1. Optical setup** ··········································································································· | **2**· |
| --- | --- |
| **Figure S2. The fluorescence variation between fluorescence particles (*N* = 236)** ·· | **3**· |
| **Figure S3. Difference image and fitting overlapped ring patterns** ······························· | **4**· |
| **Figure S4. Acquisition of the relative axial position (z_r_)** ·················································· | **5**· |
| **Figure S5. Positioning of nanoslits using bright-field image** ··········································· | **6**· |
| **Figure S6. Lateral and axial distance of fluorescence bead** ············································· | **7**· |
| **Figure S7. Correction factor for axial localization of fluorescence** ······························· | **8**· |
| **Figure S8. Relationship of input laser power vs. fluorescence intensity** ······················ | **11**· |
| **Figure S9. Photobleaching test** ································································································· | **12**· |
| **Figure S10. Individual near-field map obtained by particle 11-14** ······························· | **13**· |
| **Figure S11. Lateral and axial velocity of fluorescent nanoparticles 1-14.** ·················· | **14**· |
| **Figure S12. Near-field distribution with 488 nm and 515 nm of wavelength** ············ | **15** |
| **Figure S13. Lateral and axial localization precision map** ·············································· | **16** |
| **Figure S14. Individual near-field map of nanoslits (*w* = 100 and 200 nm)** ·················· | **17**· |
| **Figure S15. Effect of emitter size** ····························································································· | **18**· |
| **Supplementary Text : Calibration of diffraction ring pattern** ······································· | **19**· |

**Figure S1**

**Optical setup**


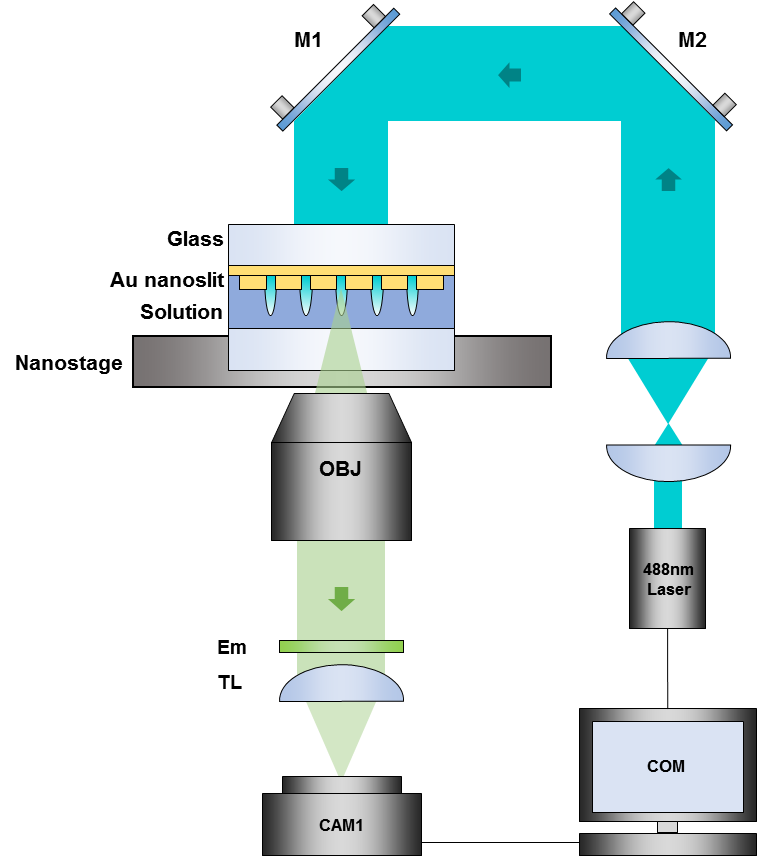


Excitation light from 488-nm diode laser was expanded by a beam expander with light incident perpendicular on the gold nanoslits. The position of gold nanoslits was adjusted in 3D by the combination of a microscope motor stage and a nanostage. Emission light from fluorescent nanoparticles was filtered by emission filter and collected by an objective lens. An electron multiplying charge-coupled device was used to capture light emission with a 2X magnification changer.

**Figure S2**

**The fluorescence variation between fluorescence particles (*N* = 236)**


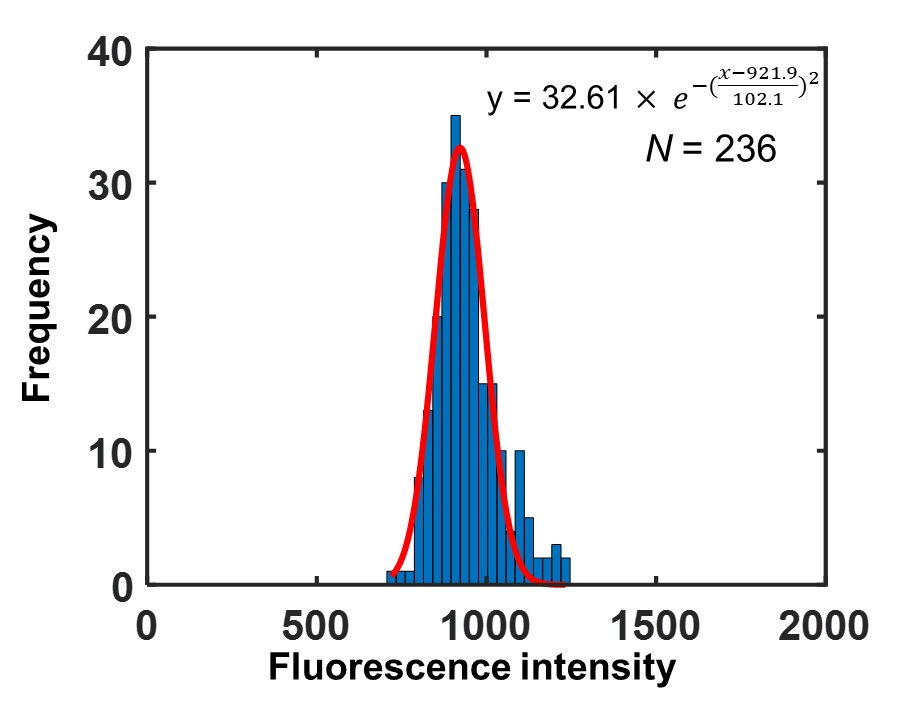


The fluorescence intensity variation between fluorescence particles was tested (*N* = 236). The mean and standard deviation was calculated to be 959.2071 and 92.6538, respectively.

**Figure S3**

**Difference image and fitting overlapped ring patterns**

For the overlap of moving fluorescence beads were analyzed using the following procedure.

Note that n and p are the indices of frame and nanoparticle.

When two nanoparticles are overlapped, the intensity came from the other nanoparticle rings should be removed for obtaining fluorescence intensity defined by an average of the brightest 100. Figure S1 (f) is the reconstructed ring image by fitting the ring images of Fig. S1(e). Using fitted variables, each ring images could be reconstructed as shown in Fig. S1(g). Simple subtraction the raw image (Fig. S1(e)) from the reconstructed image (Figure. S1(g)) enables the formation of single ring images (Fig. S1(h)) as there is no overlap with other nanoparticles. Therefore, even though there is the overlap of two nanoparticles, fluorescence intensity and axial position were successfully obtained like ring images without overlap.

**Figure S4**

**Acquisition of the relative axial position (z_r_)**


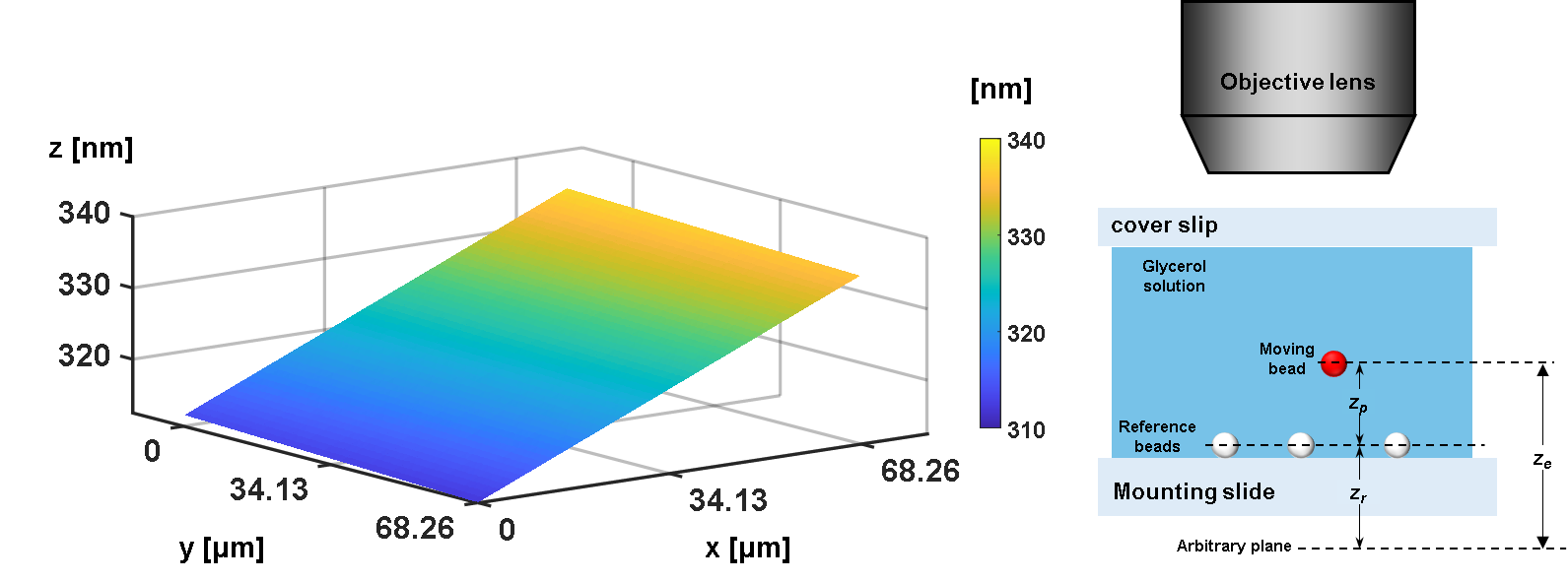


In order to obtain the relative z position of moving fluorescence bead, the z position of substrate surface is needed, i.e., the relative z position is the distance between fluorescence bead and substrate surface. The diffraction ring patterns of stuck beads at the surface was analyzed as reference positions. The imaginary planar surface formed by the three-dimensional position of 3 points was obtained as equation. The z position substrate under moving fluorescence bead can be obtained by plugging x and y coordinate in the equation. The relative axial position of fluorescent emitters from the arbitrary plane surface *z_r_* was then obtained by subtracting the position of substrate surface *z_p_* from that of fluorescent emitters *z_e_*. Here, the arbitrary plane represents a focal plane with an arbitrary focal distance.

**Figure S5**

**Positioning of nanoslits using bright-field image**

The registration of nanoslit position was implemented by fitting the bright-field image to sinusoidal equations. Two lines (red and blue) along y direction near fluorescence bead trajectories are selected and bright-field intensity profiles along the lines were fitted to sinusoidal function with the same periodicity with nanoslits, *y* = *a* + *b* sin(2*π*(*x*+*c*)/*Λ*). The periodic peaks of two lines are connected and the coordinates nanoslits are finally found as yellow lines.

**Figure S6**

**Lateral and axial distance of fluorescence bead**


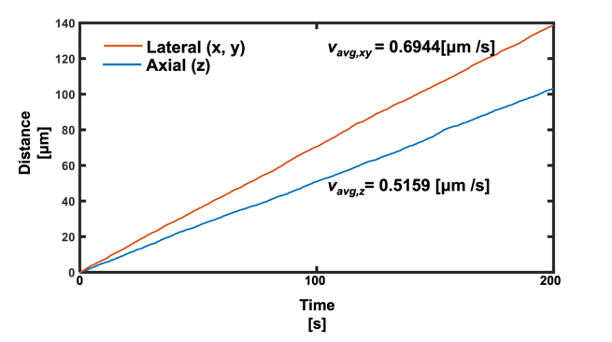


The lateral and axial distance of a fluorescent bead emitter are calculated for 200 seconds. The average lateral and axial velocity were 0.6944 and 0.5159 [μm/s], respectively.

**Figure S7**

**Correction factor for axial localization of fluorescence**


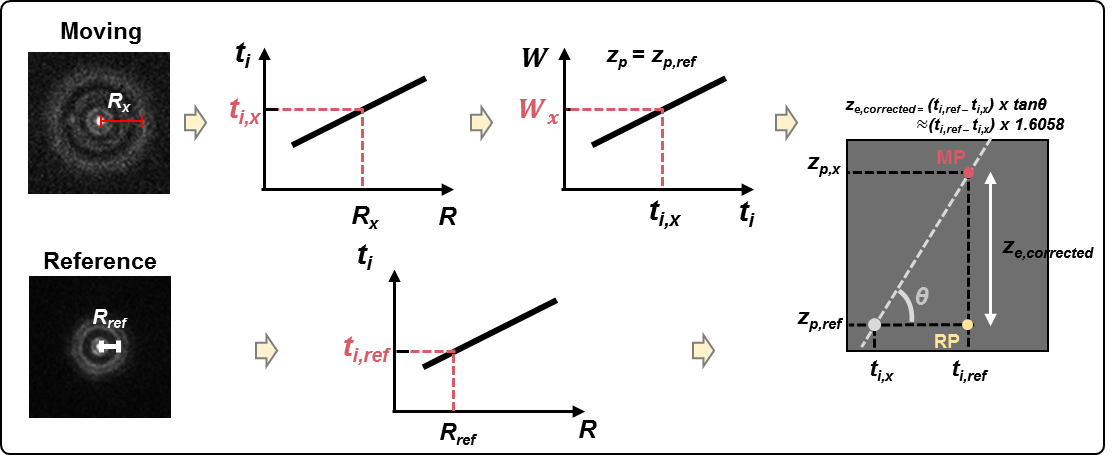


The intensity of PSF (*I_PSF_*) at a detector point (*x_d_*, *y_d_*, *z_d_*) can be obtained as

$I_{PSF}={(\frac{ka^{2}A_{0}}{z_{d}^{2}})}^{2}\left| \int_{0}^{1} J_{0}\left[ k\alpha\rho\frac{{(x_{d}^{2}+y_{d}^{2})}^{1/2}}{z_{d}} \right] exp\left[ jW \right] \rho d\rho\right|^{2}$, (S6.1)

where *k* and *W* denote an angular wave number and optical path length (OPL) difference. *a* is the radius of a circular aperture, and *ρ* is the normalized radius in the back focal plane of objective lens. If *W* is zero, an image represents the well-known Airy disk pattern and a ring pattern otherwise, while the outermost ring radius is determined by *W*.

The optical path of *W* can be represented by

$W=kn_{s}z_{p}\sqrt{1-{(\frac{NA\rho}{n_{s}})}^{2}}+kn_{i}t_{i}\sqrt{1-{(\frac{NA\rho}{n_{i}})}^{2}}+\frac{ka^{2}(z_{d}^{*}-z_{d})}{2z_{d}^{*}z_{d}}\rho^{2}$, (S6.2)

assuming that nominal and actual refractive indices of immersion layer are equal [1]. Here *n_s_* and *n_i_* denote the refractive index of sample and immersion layer. *z_p_* and *z_d_* represent the distance between particle and cover slip and the distance between detector and back focal plane of an objective lens, respectively. Note that $z_{d}^{*}$ is the nominal value of *z_d_*. *t_i_* denotes the thickness of an immersion layer.

In this experiment, *z_d_^*^* is equal to *z_d_* because the position of the detector and the back focal plane is fixed. Therefore, the third term of *W* becomes zero. With these assumptions, *W* can be approximated by a Maclaurin series as follows:

$W=W_{\rho=0}+\sum_{n=1}^{\infty} \frac{W^{\left( n \right)}(0)}{n!}\rho^{n}= k\left( n_{s}z_{p}+n_{i}{\Delta t}_{i} \right)+f\left( \rho\right)$, (S6.3)

where $C_{0}=\left( {ka^{2}A_{0}}/{z_{d}^{2}} \right)^{2}$ and a higher-order correction term in the OPL $f\left( \rho\right)=\sum_{n=1}^{\infty} \frac{W^{\left( n \right)}\left( 0 \right)}{n!}\rho^{n}$. *I_PSF_* can be expressed as

$I_{PSF}=C_{0}\left| \int_{0}^{1} J_{0}\left[ k\alpha\rho\frac{\left( x_{d}^{2}+y_{d}^{2} \right)^{\frac{1}{2}}}{z_{d}} \right] exp\left[ jk\left( n_{s}z_{p}+n_{i}{\Delta t}_{i} \right)+jkf\left( \rho\right) \right] \rho d\rho\right|^{2}$ $= C_{0}\left| \int_{0}^{1} J_{0}\left[ k\alpha\rho\frac{\left( x_{d}^{2}+y_{d}^{2} \right)^{\frac{1}{2}}}{z_{d}} \right] exp\left[ jkf\left( \rho\right) \right] \rho d\rho\right|^{2}$ (S6.4)

*t_i_* represents the immersion medium thickness. Movement of an objective lens causes *t_i_* to change (Δ*t_i_*). *z_p_* is the position associated with a moving emitter (see Section 2.2). $f\left( \rho\right)$ needed in Eqs. (S6.3) and (S6.4) can be determined by the variables of *k*, NA, *n_i_*, *n_s_*, Δ*t_i_*, and *z_p_*. In order to find a set of [*t_i_*, *z_p_*] under the constraint of identical *f*(*ρ*), we have defined *L* to measure the similarity of *f*(*ρ*) with regard to the sets of [*t_i_*, *z_p_*]:

$L\left( \left[ t_{i}^{a},z_{p}^{a} \right],\left[ t_{i}^{b},z_{p}^{b} \right] \right)= \int_{0}^{1} \left| f^{a}\left( \rho\right)-f^{b}\left( \rho\right) \right|d\rho$, (S6.5)

assuming that $f^{a}\left( \rho\right)$ = $f\left( \rho\right)$ when ${[t}_{i},z_{p}]$ = [$t_{i}^{a},z_{p}^{a}$].

The above figure illustrates the procedure for correction of the axial position of a moving emitter based on relationships between optical path difference and [*t_i_*, *z_p_*]. The radius of a moving emitter and reference can be measured by fitting with Gaussian function. The z position of a moving emitter is denoted as *z_p,ref_* which is assumed to be identical to that of a reference emitter. How much *t_i_* needs to be changed to obtain the corresponding radius can be determined by the calibration curve which was measured in Figure 2(b). The corresponding *t_i_* is *t_i,x_*. Note that the radius of a moving emitter changes instead of Δ*t_i_*, because of the variation in z position. Therefore, it is necessary to locate a point *t_i_* = *t_i,ref_* among those that correspond to the same ring radius with the point [*t_i,x_*, *z_p,ref_*] to find *z_p_* of the moving emitter. In other words, we locate a point *t_i_* = *t_i,ref_* among the points with the same W values. As shown in Figure 4(b, c), the contour is a line with a slope of about 1.6058. Among the points on the line passing through (*t_i,x_*, *z_p,ref_*) with the slope, the point corresponding to *t_i_* = *t_i,ref_* represents the moving emitter. Finally, we can correct the position of a moving emitter relative to the reference bead by multiplying (*t_i,ref_* – *t_i,x_*) by *tanθ*. In the text, we define the multiplicative factor *tanθ* as the correction factor (*cf*).

**Reference**

[1] Hell, S.; Reiner, G.; Cremer, C.; Stelzer, E.H. Aberrations in confocal fluorescence microscopy induced by mismatches in refractive index. *J. Microsc.* **1993**, 169, 391-405.

**Figure S8**

**Relationship of input laser power vs. fluorescence intensity**


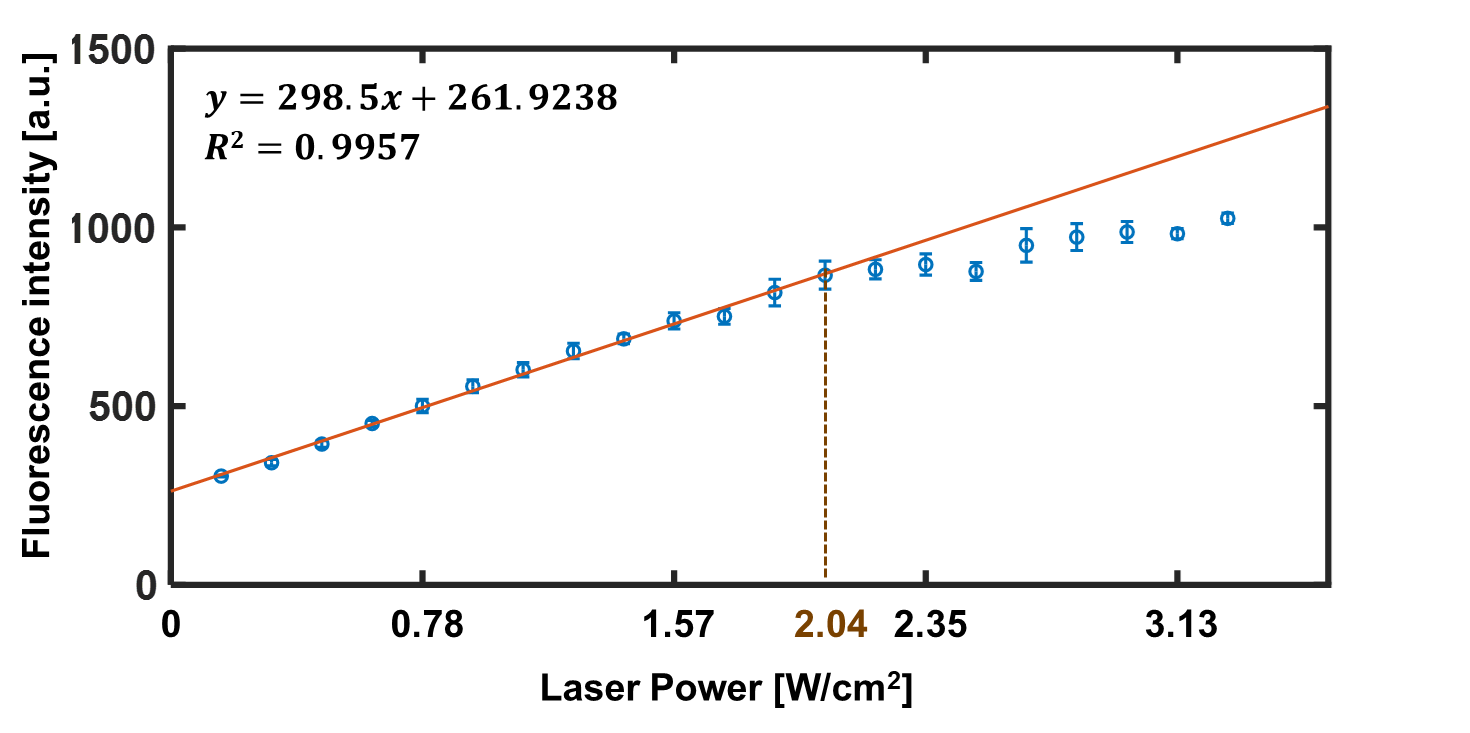


The linearity between fluorescence intensity and input laser power was tested. The correlation coefficient R^2^ was calculated to be 0.9957 in the range of input laser power 0 ~ 2.04 W/cm^2^. In other words, fluorescence intensity is linear with input laser power in this range. FDTD results near nanoslits suggest that the maximum near-field intensity to be smaller than that of incident light. With maximum optical power density set at 2.04 W/cm^2^, the near-field intensity obtained from nanoslits should be in the linear range.

**Figure S9**

**Photobleaching test**

In order to test photobleaching, we measured fluorescence intensity of diffraction ring patterns from 3 emitter particles for the duration of 200 seconds. Although there was fluctuation in the fluorescence intensity, the mean value for the first 20 seconds and the last 20 seconds showed difference smaller than 1%. The test was performed using excitation light with optical power density of 2.0 W/cm^2^ (nominal) or 1.3 W/cm^2^ obtained from the normalization of maximum optical power density. The results indicate little fluorescence photobleaching in the course of diffraction ring pattern imaging because image acquisition was performed for less than or equal to 200 seconds per emitter particle.

**Figure S10**

**Individual near-field map obtained by particle 11-14**


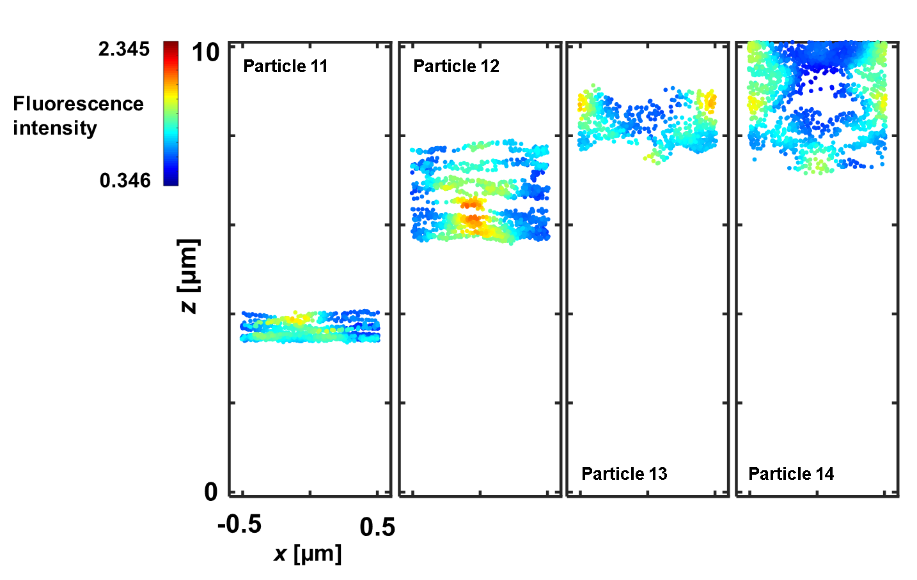


Individual near-field maps in the xz plane obtained by particle 11-14. The near-field map in Figure 5b can be constructed by merging individual near-field maps of particle 1-14.

**Figure S11**

**Lateral and axial velocity of fluorescent nanoparticles 1-14.**

| # | Average lateral velocity (μm/s) | Average axial velocity (μm/s) | Measured time ($s$) |
| --- | --- | --- | --- |
| 1 | 0.694429 | 0.515914 | 200 |
| 2 | 0.629458 | 0.528975 | 200 |
| 3 | 0.661853 | 0.457017 | 200 |
| 4 | 0.238831 | 0.217186 | 152.7 |
| 5 | 0.608111 | 0.522768 | 190.8 |
| 6 | 0.652497 | 0.493349 | 199.2 |
| 7 | 0.268136 | 0.167452 | 804 |
| 8 | 0.644457 | 0.555815 | 199.1 |
| 9 | 0.595321 | 0.39913 | 200.0 |
| 10 | 0.675494 | 0.456493 | 198.9 |
| 11 | 0.152737 | 0.177315 | 118.5 |
| 12 | 0.197992 | 0.189557 | 162 |
| 13 | 0.567777 | 0.383355 | 173.1 |
| 14 | 0.219741 | 0.154803 | 66.5 |

The velocity of fluorescent nanoparticle emitters in the lateral plane and along the axial direction is calculated. Average lateral velocity ranged from 0.152737 to 0.694429 (μm/s) while average axial velocity was in the range from 0.154803 to 0.555815 (μm/s). Note that the frame rate was 10 frame/second. The average lateral velocity tends to be higher than the average axial velocity mostly in association with solution drift, i.e., without any special microfluidic channels or pumps to control the flow, the capillary force in the solution between the glass plates may induce more active movement of fluorescent emitters in the lateral dimension.

**Figure S12**

**Near-field distribution with 488 nm and 515 nm of wavelength**


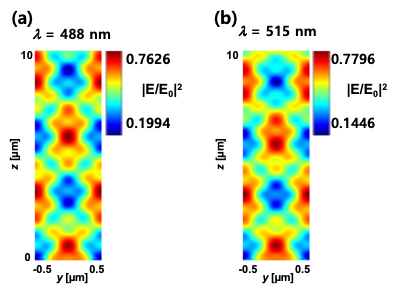


3D near-field intensity distribution near nanoslits was calculated both at excitation and emission wavelength (λ = 488 nm and 515 nm). The near-field distribution at 515 nm appears axially shifted from the one at 488 nm due to the dispersion of sample medium without significant difference in the maximum and minimum intensity.

**Figure S13**

**Lateral and axial localization precision map**


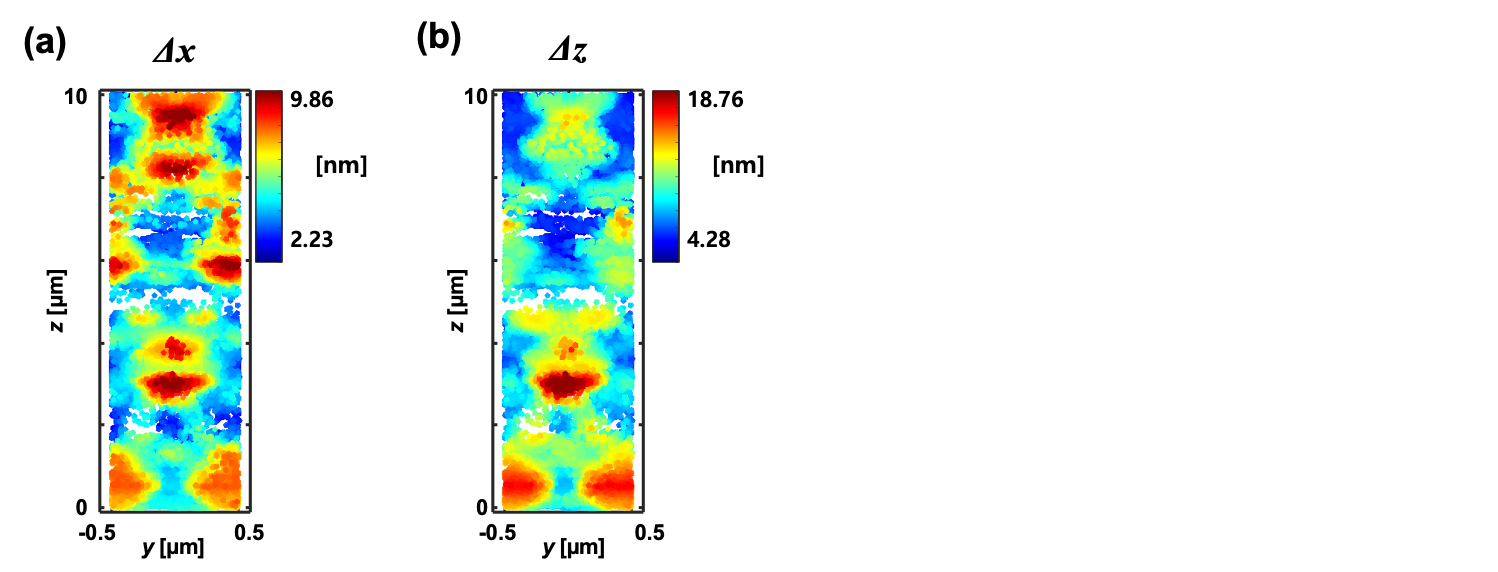


The lateral and axial localization precision was presented in (a) and (b), respectively. The axial precision (Δz) is roughly twice as large as the one in the lateral plane (Δx). The results are based on Figure 2(g-l) and Figure 5(b) and do not include the localization error associated with blurred image defocus caused by the displacement of an emitter per frame.

**Figure S14**

**Individual near-field map of nanoslits (*w* = 100 and 200 nm)**


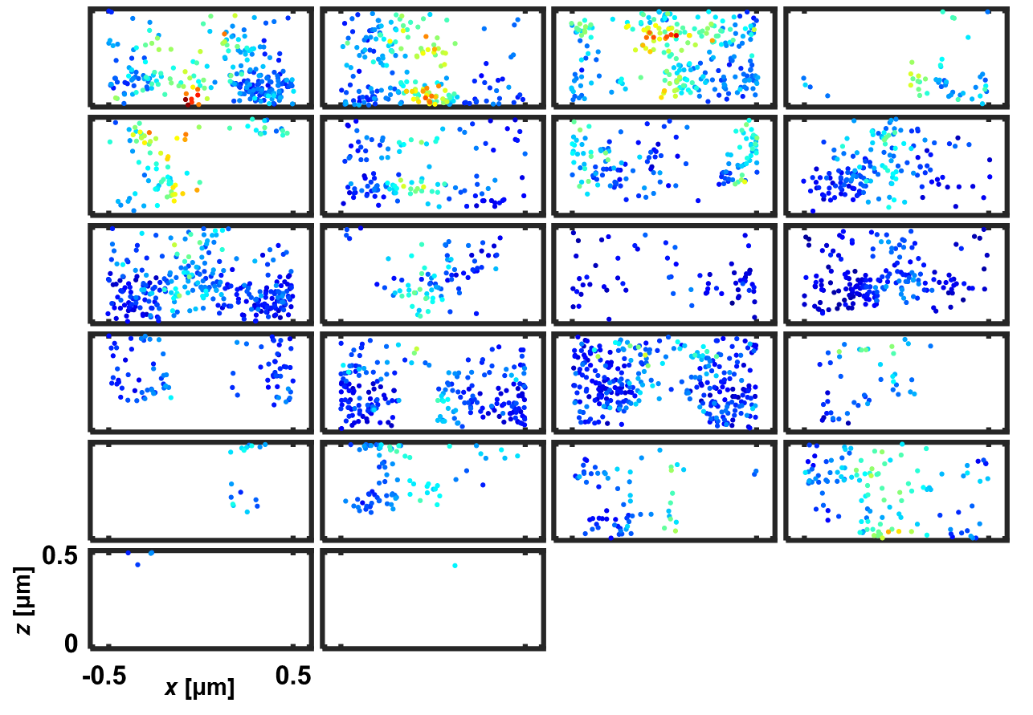


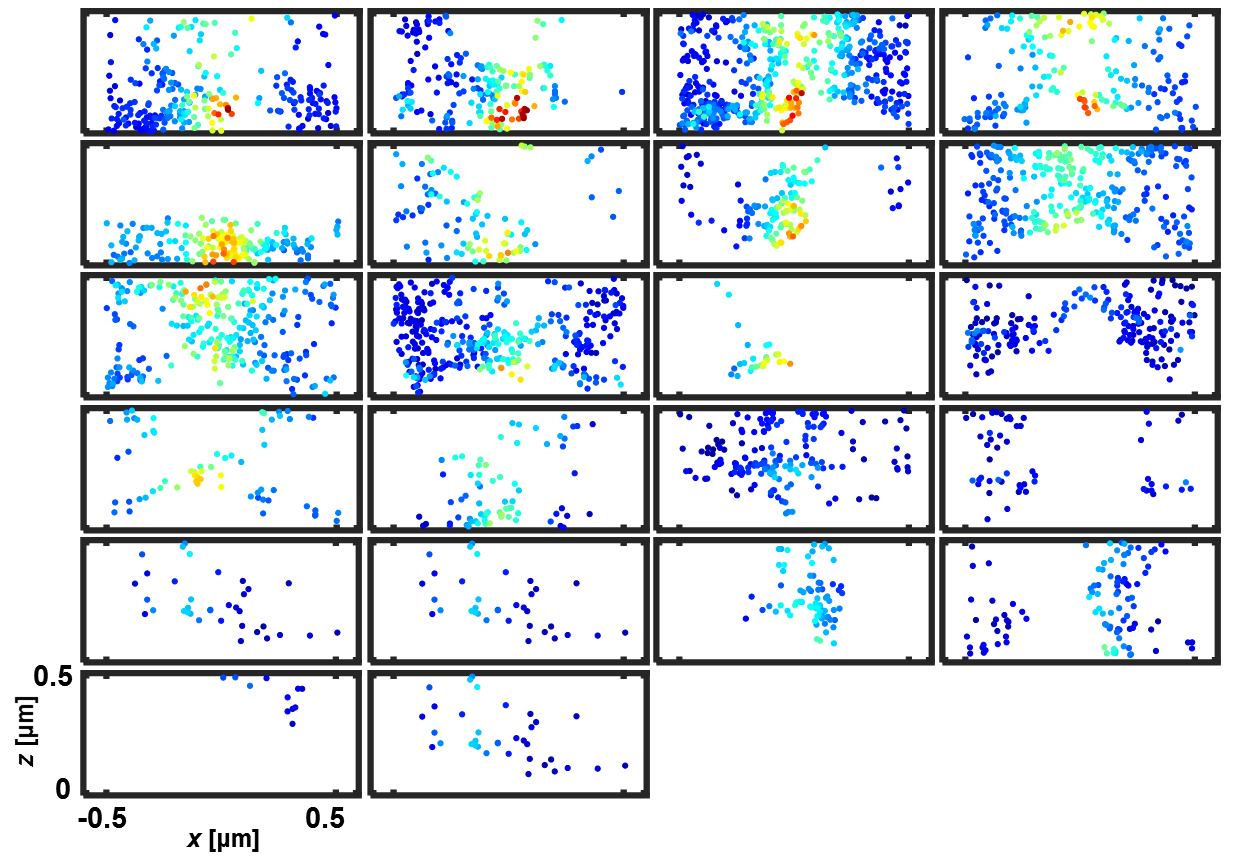


Individual near-field map of 200-nm (top) and 100-nm (bottom) nanoslits in the yz plane obtained by fluorescence particles.

**Figure S15**

**Effect of emitter size**


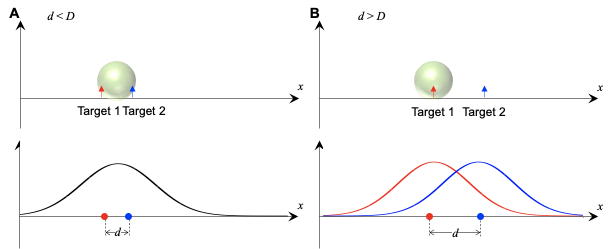


The resolution of DePLOMA is dependent directly on the size of fluorescent emitters. Here we assume that targets are delta function like dots but have optical field intensity. If the emitter size D is smaller than the distance of two targets, the fluorescence emitter is excited at the same. On the other hand, the moving fluorescence emitter can be excited by each target independently, so it is possible to distinguish two targets.

**Supplementary Text**

**Calibration of diffraction ring pattern**

The diffraction pattern of single bead is changed depending on its axial position as shown in Fig. 2a. 6 representative images show concentric rings sharing same center, and the number and radius of ring are changed by z position. Note that the axial positions do not indicate distance from focal plane, but the relative position from the plane the first ring appears. In order to obtain the relationship between diffraction pattern and axial position, the ring patterns are fitted to the function F, the function composed of Gaussian function at center and concentric Gaussian rings sharing the same center with different radiuses and widths, and the radius of outermost ring is used for axial position calibration. The sample for calibration is prepared by dried the 200-nm bead solution on glass coverslip to fix them. Fluorescence bead images were capture at every 100-nm axial displacement using nanostage. For each axial position, 15 images were captured, and exposure time was fixed to 100 ms. Fig. 2b presents the calibration curve, in which the relationship between the outermost ring radius and the relative axial position is monotonically increasing and the calibration curve was fitted to 2th order polynomial function and plotted as red line.

1. These authors equally contributed to this work. [↑](#footnote-ref-2)
2. Current affiliation: Center for Systems Biology, Massachusetts General Hospital, Boston, Massachusetts, USA [↑](#footnote-ref-3)
3. Current affiliation: LG Display, Paju, Gyeonggi-do, Korea, 10845 [↑](#footnote-ref-4)
